# pH-Responsive Nanogels from Bioinspired Comb-like Polymers with Hydrophobic Grafts for Effective Oral Delivery

**DOI:** 10.3390/gels11100806

**Published:** 2025-10-08

**Authors:** Qinglong Liu, Dewei Ma, Haoze Cheng, Keke Yang, Bo Hou, Ziwen Heng, Yu Qian, Wei Liu, Siyuan Chen

**Affiliations:** 1 Research Institute for Biomaterials, Tech Institute for Advanced Materials Bioinspired Biomedical Materials & Devices Center, College of Materials Science and Engineering, Jiangsu Collaborative Innovation Center for Advanced Inorganic Function Composites, Suqian Advanced Materials Industry Technology Innovation Center, Nanjing Tech University, Nanjing 210037, China; 2 College of Life Science and Technology, Huazhong University of Science and Technology, Wuhan 430074, China

**Keywords:** pH-responsive, nanogel, comb-like, anionic polymer, oral delivery

## Abstract

Oral administration remains the most patient-friendly drug delivery route, yet its efficacy is limited by physiological barriers including gastric degradation and inefficient cellular uptake. pH-responsive nanogels have shown promise for gastrointestinal drug delivery, though their effectiveness is often constrained by poor membrane interaction. Inspired by natural membrane-anchoring mechanisms, a series of comb-like anionic polymers were designed via grafting alkylamines of different chain lengths (C_10_, C_14_, C_18_) at varying densities (10–30%) onto a biodegradable poly(L-lysine isophthalamide) (PLP) backbone. These pH-responsive comb-like polymers self-assembled into nanogels for loading the hydrophobic chemotherapeutic agent camptothecin. The alkyl length and grafting density significantly influenced pH-responsive behavior, membrane disruption, and drug release profiles. The optimal formulation—the nanogel prepared with PLP grafted 30% C_14_—achieved a high drug-loading capacity, ideal particle size and stability, and offered superior protection in acidic conditions (only 7 ± 5% release at pH 1.2 over 24 h), while enabling rapid intestinal release (78 ± 2% at pH 7.4 within 24 h). The nanogels significantly enhanced cellular uptake, cytoplasmic delivery, and cytotoxicity against colorectal carcinoma cells. This study demonstrates the key role of hydrophobic modification in designing effective oral nanocarriers, providing a promising platform for the treatment of intestinal diseases.

## 1. Introduction

Oral administration is the primary and most convenient route for drug delivery. However, the gastrointestinal tract presents significant physiological barriers that compromise therapeutic efficacy [1,2,3]. The highly acidic gastric environment (pH 1.2–3.0) can induce drug degradation and premature release, substantially reducing the bioavailable dose reaching intestinal absorption sites [4,5,6]. Subsequent cellular uptake introduces further challenges [7,8], as therapeutic agents entrapped within acidic endosomes and lysosomes (pH 5.0–6.5) are susceptible to enzymatic degradation unless they escape promptly, ultimately diminishing therapeutic outcomes [9,10]. Consequently, the development of oral delivery systems that can respond to gastrointestinal pH gradients, protect payloads, and promote endosomal escape is critical for advancing oral therapeutics.

pH-responsive nanogels, fabricated from pH-sensitive polymers, have emerged as promising candidates for oral drug delivery. As nanosized hydrogel particles, nanogels combine the advantages of both hydrogels and nanoparticles, making them attractive as targeted nanocarriers for controlled release and enhanced drug stability in nanomedicine. These viscoelastic materials consist of hydrophilic polymer networks in the sub-micron size range and exhibit favorable properties such as biocompatibility, high stability, tunable particle size, and responsiveness to external stimuli [11]. pH-responsive nanogels, in particular, undergo swelling or collapse due to protonation or deprotonation of ionizable groups within the polymer network. While cationic pH-responsive polymers may disrupt cell membranes through electrostatic interactions with anionic phospholipids—potentially causing membrane permeabilization or lysosomal rupture—anionic pH-responsive polymers offer improved safety profiles for nanogel design [12,13].

The negative charge of anionic polymers reduces nonspecific interactions with the negatively charged cell membrane surface, thereby minimizing membrane damage and cytotoxicity. This makes anionic pH-responsive nanogels more suitable for long-term in vivo applications. Common examples include polymers bearing carboxylic acid groups, such as poly(methacrylic acid) (PMAA) [14,15] and poly(propyl acrylic acid) (PPAA) [16]. Below their p*K*_a_, these anionic polymers undergo protonation of carboxylate groups, reducing electrostatic repulsion within the network and leading to colloidal collapse [17,18,19]. This pH-dependent behavior is advantageous for oral delivery: under acidic gastric conditions, protonation promotes a hydrophobic, collapsed state that shields encapsulated drugs from degradation, whereas deprotonation in the neutral intestine enhances hydrophilicity and facilitates drug release. Furthermore, the pH-triggered conformational transition can expose hydrophobic segments that promote membrane fusion or disruption, aiding endosomal escape [20]. However, the inherent negative charge also impedes interaction with cellular and endosomal membranes, limiting cellular uptake and compromising therapeutic efficacy. Thus, there is a need to enhance the cellular interactions of anionic nanogels.

In nature, many membrane proteins incorporate covalently attached long-chain fatty acids that serve as hydrophobic anchors, embedding into lipid bilayers to strengthen membrane binding. Often, multiple hydrophobic moieties are present to ensure robust membrane association. Inspired by this mechanism, our group previously conjugated long aliphatic chains onto a pH-responsive, anionic poly(L-lysine isophthalamide) (PLP) backbone, forming a comb-like architecture to augment polymer–membrane interactions. PLP—synthesized from biocompatible L-lysine methyl ester dihydrochloride and isophthaloyl chloride—is a biodegradable, pH-responsive polymer [21,22,23]. However, its membrane activity is limited to relatively low pH values. By grafting decylamine (NDA) at 18 mol% onto the side-chain carboxylic groups of PLP, we developed a comb-like polymer that exhibited negligible hemolytic activity at physiological pH (7.4) but induced rapid membrane disruption within 20 min at endosomal pH (5.5), enabling efficient cytosolic delivery across multiple cell lines [21]. Although these findings underscore the importance of hydrophobic modification in enhancing membrane interactions, the effects of alkyl chain length and grafting density on biocompatibility, drug loading, and in vivo performance remain inadequately explored and require systematic investigation.

**Figure 1:**
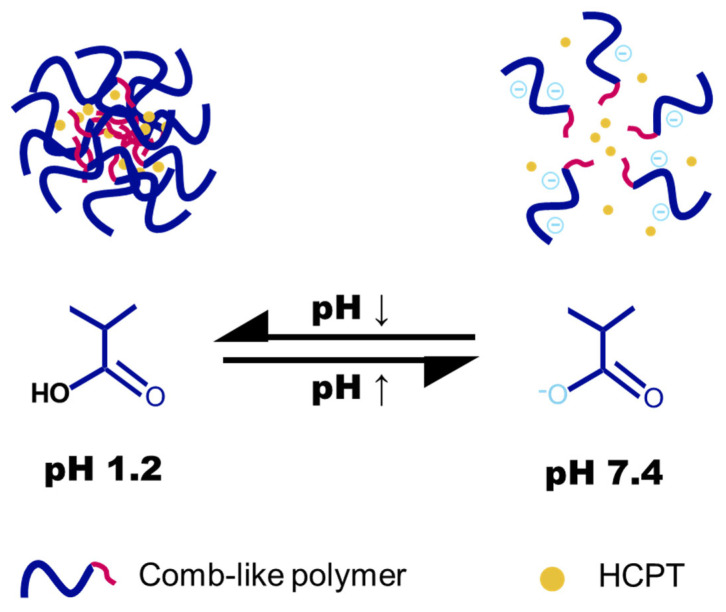
Schematic illustration of the pH-dependent drug release from nanogels formed by comb-like polymers bearing hydrophobic grafts.

In this study, we developed a pH-responsive nanogel system with pH-modulated membrane activity to promote active intestinal absorption and efficient release of a hydrophobic chemotherapeutic drug (Figure 1). This nanogel is engineered with a hydrophobic core, which is expected to provide enhanced drug-loading capacity for the hydrophobic agent. A series of comb-like polymers were synthesized with varying alkyl chain lengths (C_10_, C_14_, C_18_) and grafting densities (10–30%). Nanogels were prepared through physical crosslinking of these polymers and loaded with the model hydrophobic drug camptothecin (HCPT). Under acidic gastric conditions (pH 1.2–3.0), the nanogels remain collapsed, protecting HCPT from degradation. Upon entry into the neutral intestinal environment, carboxylate deprotonation triggers swelling and rapid drug release. We investigated the influence of alkyl chain length and grafting density on the pH-responsive phase transition and membrane disruption efficacy. The size, stability, encapsulation efficiency (EE), and drug-loading (DL) capacity of nanogels derived from different polymer variants were compared to identify an optimal formulation. Additionally, cellular uptake, in vitro cytotoxicity, and antitumor potency against human colorectal carcinoma cells were evaluated.

## 2. Results and Discussion

### 2.1. Structural Characterization of pH-Responsive Comb-like Polymers

**Figure 2:**
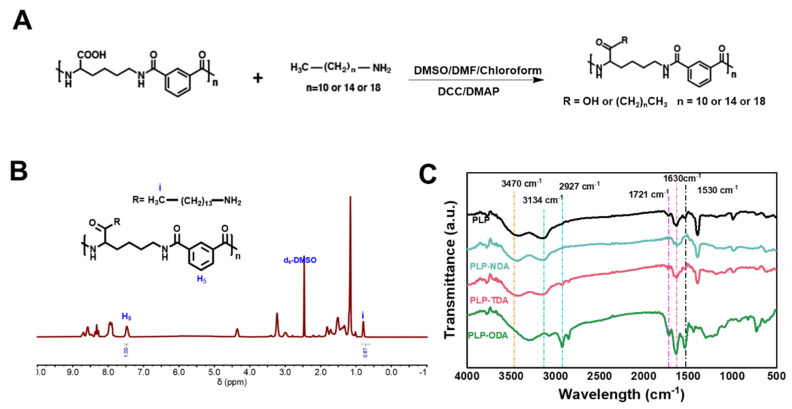
Synthesis and characterization of PLP-based polymers. (**A**) Synthetic route for PLP and alkylamine-grafted PLP. (**B**) 1H NMR spectrum of PLP-TDA 30%. (**C**) FT-IR spectra of PLP and alkylamine-grafted PLP with varying chain lengths.

Anionic pH-responsive comb-like polymers exhibiting tunable membrane activity and switchable charge properties were successfully synthesized via Steglich esterification [24]. This reaction established covalent bonds between the PLP backbone and hydrophobic alkylamines (C_10_, C_14_, C_18_) at defined grafting densities (10%, 20%, 30%), following the synthetic route depicted in Figure 2A. Copolymer synthesis and structural integrity were initially confirmed by ^1^H NMR spectroscopy. Quantitative analysis of the integration ratios between methyl protons (0.77–0.91 ppm) and aromatic protons (7.45–7.64 ppm) enabled precise determination of grafting density, as evidenced in Figure 2B and Supplementary Figures S1–S3. These spectra confirmed the effective introduction of alkylamine moieties while preserving the main chain structure.

Complementary Fourier transform infrared (FT-IR) analysis (Figure 2C) further validated the synthesis. Key spectral features included a broad peak at 3470 cm^−1^ (N-H stretching from lysine amino groups and amide bonds, indicating hydrophilic interactions), a peak at 3134 cm^−1^ (confirming the PLP backbone), a carbonyl peak at 1724 cm^−1^ coupled with a weakened carboxylic acid peak at 1721 cm^−1^ and characteristic amide I/II bands at 1630 cm^−1^ and 1530 cm^−1^ (collectively signifying amide bond formation). The intensity of alkyl chain C-H stretching vibrations at 2927 cm^−1^, proportional to grafting density, confirmed hydrophobic-side-chain incorporation. Unaltered backbone amide peaks indicated preservation of the polymer backbone during modification [25]. ^1^H NMR and FT-IR results robustly establish the successful synthesis of PLP-alkylamine copolymers with the intended chemical structure and modification efficiency, providing a solid foundation for subsequent functional characterization.

### 2.2. Aqueous Solution Properties

**Figure 3:**
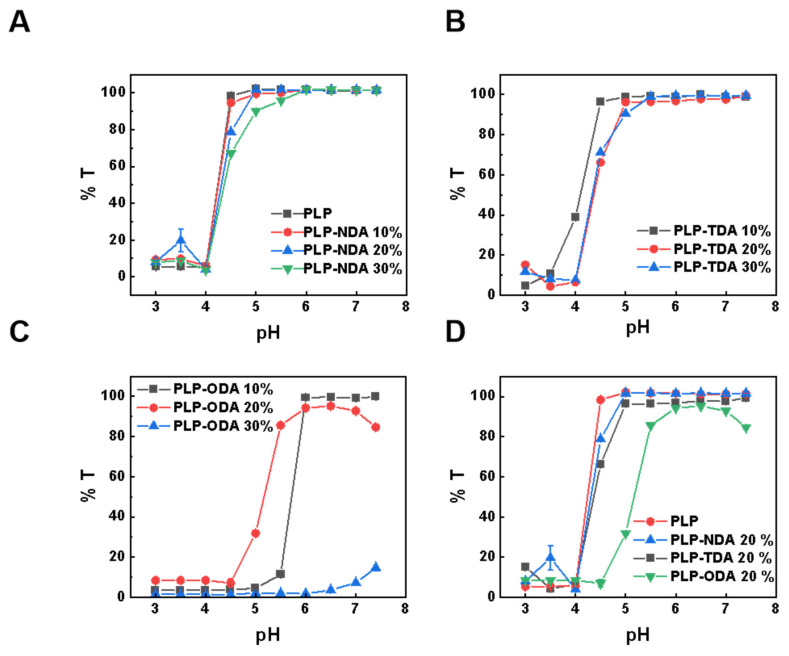
Transmittance of polymers at a concentration of 0.5 mg/mL. pH-dependent transmittance of (**A**) PLP-NDA, (**B**) PLP-TDA, and (**C**) PLP-ODA at different grafting rates. (**D**) Transmittance of polymers with identical grafting rates (20%) but varying alkyl chain lengths at different pH values.

The pH-dependent phase separation behavior and particle size dynamics of comb-shaped PLP-NDA, PLP-TDA (PLP-tetradecylamine), and PLP-ODA (PLP-octadecylamine) copolymers bearing long hydrophobic side chains were quantitatively analyzed [26]. In aqueous solution, the polymers are soluble and molecularly dispersed at neutral pH, resulting in high transmittance (close to 100%). As the pH decreases, the carboxylic acid groups along the polymer backbone become progressively protonated, reducing electrostatic repulsion and enhancing the driving force for hydrophobic association. When a critical pH (the precipitation onset pH, pH_p_) is reached, this triggers polymer aggregation and phase separation, causing the solution to turn turbid. This turbidity is quantitatively measured as a sharp decrease in transmittance. As shown in Figure 3A, increasing the NDA grafting density from 10% to 30% elevated the pH_p_ from 4.0 (PLP) to 5.0, indicating enhanced polymer hydrophobicity shifting phase separation to higher pH. Similar trends were observed for PLP-TDA (Figure 3B) and PLP-ODA (Figure 3C), confirming the synergistic effect of side-chain hydrophobicity and grafting density on pH_p_. Notably, PLP-ODA at 30% grafting density exhibited poor aqueous solubility at neutral pH, signifying that strong hydrophobic interactions dominated over charge repulsion. Consistent with Figure 3D and Table S2, at equivalent grafting densities (20%), longer alkyl chains (C18, ODA) progressively increased pH_p_ from 4.5 to 5.5, attributed to enhanced intermolecular hydrophobic association promoting conformational changes at higher pH. Similar trend could be observed in the critical aggregation concentration (CAC) value. As summarized in Table S2, the CAC value of the comb-like polymer decreased with increasing the molar percentage of alkyl chain grafts and the chain length, which could be attributed to enhanced polymer hydrophobicity.

**Figure 4:**
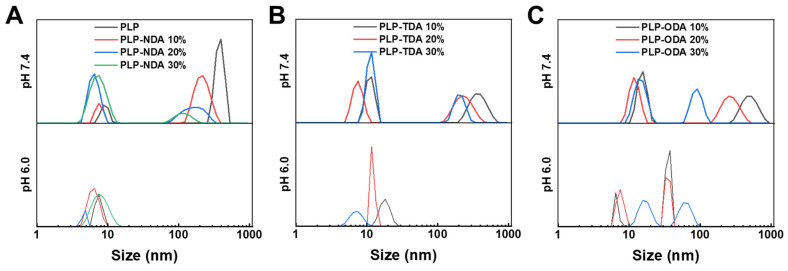
Particle size change in (**A**) PLP-NDA, (**B**) PLP-TDA, and (**C**) PLP-ODA polymers at different grafting rates at pH 7.4 and pH 6.0.

Dynamic light scattering (DLS) further characterized pH-dependent particle size changes. As seen in Figure 4A, at pH 7.4, PLP-NDA with 10% grafting displayed a bimodal distribution (12 nm and 342 nm), resulting from electrostatic repulsion of deprotonated carboxyl groups promoting chain extension and aggregate formation. Upon acidification to pH 6.0, charge neutralization allowed hydrophobic interactions to dominate, significantly reducing particle size to a unimodal distribution centered at 18.2 nm. A similar trend could be observed in PLP-NDA polymers with grafting rates of 20% and 30%. As illustrated for PLP-TDA and PLP-ODA (Figure 4B,C), the extent of size reduction increased with higher grafting densities. Furthermore, polymers with longer hydrophobic chains (e.g., PLP-ODA 30%) demonstrated enhanced aggregation at pH 7.4 with increasing grafting density, highlighting the cooperative regulation of intermolecular interactions by both chain length and grafting density. However, unlike the PLP-NDA and TDA polymers, bimodal distribution was still observed in PLP-ODA polymers at pH 6.0, possibility due to the relatively longer side chain affecting the self-assembly. These findings align with prior reports on amphiphilic copolymers [27], validating the intricate interplay between pH, molecular structure, and self-assembly behavior in these comb-shaped polymers. These results establish a clear structure–property relationship for comb-shaped PLP polymers: hydrophobic side-chain length and grafting density govern the hydrophobicity of the polymer and thus, affect the pH threshold for the conformational transition from hydrophilic coils to hydrophobic aggregates. This tunability is pivotal for designing pH-responsive nanogels [28].

### 2.3. pH-Responsive Membrane Destabilization

**Figure 5:**
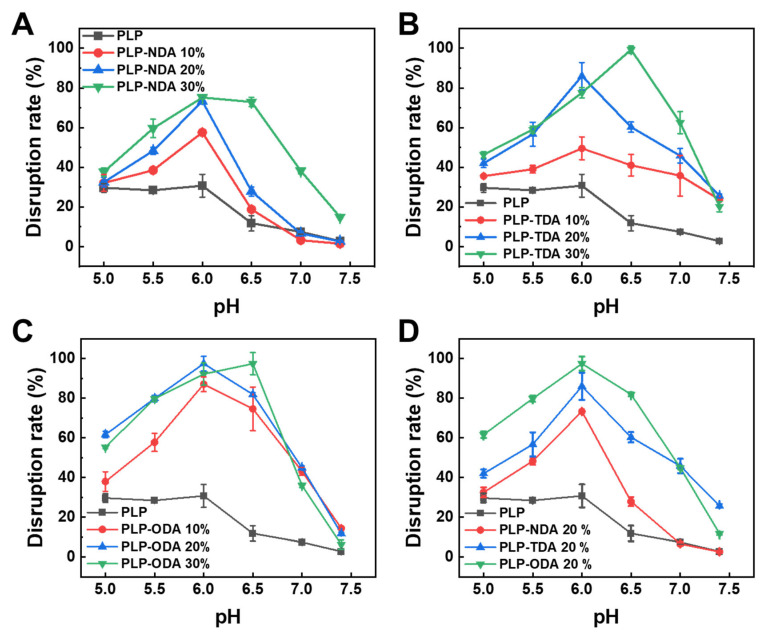
pH-responsive membrane disruption activity of PLP and alkylamine-grafted comb-like polymers on RBCs. (**A**–**C**) Relative hemolysis of (**A**) PLP-NDA, (**B**) PLP-TDA, and (**C**) PLP-ODA at varying pH values and grafting rates. (**D**) Chain length-dependent hemolysis of alkylamine-grafted PLP (20% grafting) at different pH conditions. All samples tested at identical concentrations; PLP (ungrafted) included as control in (**A**–**C**).

Red blood cell (RBC) membranes, chosen as an endosomal membrane model due to their phospholipid bilayer composition, were used to evaluate pH-responsive membrane disruption capacities across a pH range (5.0–7.4) simulating physiological to endosomal acidification [29]. Figure 5A shows that unmodified PLP induced minimal membrane disruption (30.7 ± 5.8%) only below pH 6.0. In contrast, comb-like polymers with NDA side chains exhibited negligible activity at pH 7.4 but significantly enhanced disruption upon acidification. Maximum disruption rates for PLP-NDA (10%, 20%, 30%) reached 57.6 ± 1.0%, 73.3 ± 0.4%, and 75.2 ± 0.5%, respectively, indicating that hydrophobic side chains modulate membrane interactions via enhanced insertion.

A similar trend was observed for PLP-TDA (C_14_ chains, Figure 5B). Efficacy was limited at pH 7.4 but increased with grafting density under acidic conditions (pH ≤ 6.5), achieving maxima of 50.0 ± 5.8% (PLP-TDA 10%), 86.0 ± 6.9% (PLP-TDA 20%), and 99.3 ± 1.9% (PLP-TDA 30%). Notably, for PLP-TDA 30%, the pH of maximum disruption shifted from 6.0 (pure PLP) to 6.5, demonstrating that longer hydrophobic segments enable membrane permeabilization at higher pH, broadening the effective acidic response range [30]. PLP-ODA (C_18_ chains, Figure 5C) exhibited even stronger disruption, with maxima increasing from pure PLP (30.7 ± 5.8% disruption at pH 6.0) to 87.0 ± 3.7% (PLP-ODA 10%), 97.4 ± 3.6% (PLP-ODA 20%), and 97.4 ± 5.7% (PLP-ODA 30%), directly correlating alkyl chain length with membrane-destabilizing capacity. Comparative analysis at 20% grafting density (Figure 5D) revealed a progressive increase in membrane disruption efficacy with alkyl chain length extension (C_0_~C_18_). This indicates that hydrophobic side-chain length not only strengthens polymer–membrane interactions but also regulates the pH-responsive activation threshold—longer chains at high grafting rate facilitate efficient disruption at higher pH (e.g., 6.5), while shorter chains require lower pH (e.g., 6.0).

### 2.4. Preparation and Characterization of Nanogels

pH-responsive nanogels were further fabricated from the synthesized comb-like polymers via nanoprecipitation. The particle size, polydispersity index (PDI), and storage stability over 30 days at 4 °C for these nanogels are summarized in Table 1 and Table S3. Analysis reveals a clear positive correlation between nanogel particle size and both the length of the hydrophobic side chain (C_10_-C_18_) and the grafting rates (10–30%). Specifically, for all polymer types, particle size increased progressively with higher grafting rates. Furthermore, at equivalent grafting densities, particle size increased with longer hydrophobic side chains. Nanogels derived from PLP-NDA (C_10_) and PLP-TDA (C_14_) polymers consistently exhibited low PDIs (~0.1) across all grafting densities (10–30%), indicating a narrow size distribution and high homogeneity. Critically, these nanogels demonstrated excellent stability, with particle sizes remaining virtually unchanged throughout the 30-day storage period. In contrast, nanogels prepared from PLP-ODA (C_18_) polymers displayed higher PDIs (>0.2). Notably, the PLP-ODA 30% nanogel exhibited the largest initial size (165.0 ± 13.2 nm) and a high PDI (0.37 ± 0.01). This system proved unstable during storage; after 14 days, both particle size (233 ± 62 nm) and PDI (0.5 ± 0.1) increased significantly. These results likely arose from stronger intermolecular hydrophobic interactions during self-assembly, driven by the longer alkyl chains combined with higher grafting densities, which facilitated the formation of larger aggregates. This phenomenon aligns with prior observations where enhanced polymer hydrophobicity promotes more extensive self-assembly in aqueous media [31,32].

**Figure 6:**
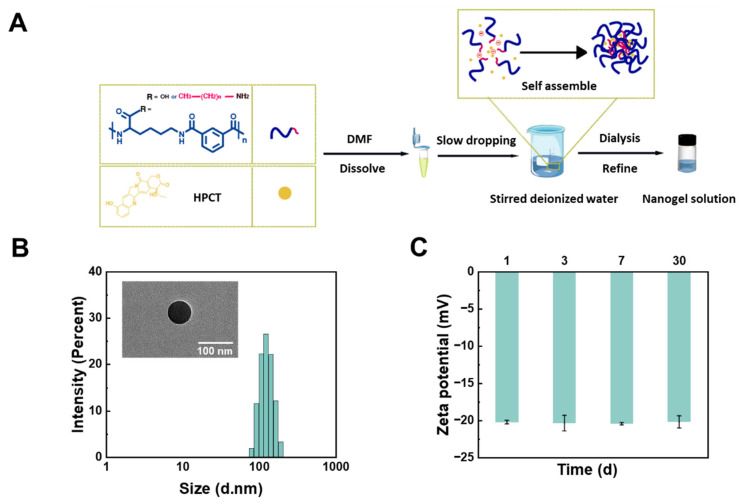
Preparation and characterization of nanogel. (**A**) Schematic illustration of the process for preparing nanogel. (**B**) Hydrodynamic size distribution (intensity-weighted) and representative TEM image of HCPT-loaded PLP-TDA 30% nanogels. (**C**) Zeta potential stability of HCPT-loaded PLP-TDA 30% nanogels stored at 4 °C for 30 days. Mean ± SD (n = 3).

The lipophilic anticancer drug HCPT was subsequently loaded into the nanogels via co-assembly with the pH-responsive comb-shaped polymer in the aqueous phase (Figure 6A). To identify the optimal nanogel formulation, the encapsulation efficiency (EE) and drug-loading capacity (DLC) of all nanogels were systematically evaluated. As shown in Table S4, HCPT loading capacity generally increased with alkylamine grafting density (10–30 mol%). This trend is attributed to the enhanced polymer hydrophobicity resulting from higher grafting densities, which strengthens hydrophobic interactions with the lipophilic drug, consequently improving both EE and DLC. However, despite possessing greater inherent hydrophobicity, PLP-ODA 30% nanogel (C_18_) exhibited a lower DLC compared with PLP-TDA 30% nanogel (C_14_). This apparent trade-off arises from the competition between two mechanisms: (1) enhanced hydrophobic drug–polymer interactions favoring encapsulation at higher grafting densities and (2) steric hindrance caused by longer alkyl chains (e.g., C_18_), which form denser networks that impede drug diffusion into the nanogel core. Within the 10–30 mol% grafting density range, the hydrophobic binding effect dominated, leading to a monotonic increase in EE. For the longest chain (C_18_), however, steric hindrance became the predominant factor, ultimately reducing DLC [33]. Considering pH-responsiveness and drug encapsulation, PLP-TDA 30% nanogel was selected as the optimal formulation for further investigation.

Following HCPT loading, the hydrodynamic diameter of HCPT-loaded PLP-TDA 30% nanogel increased slightly from 105.4 ± 1.8 nm to 128.9 ± 1.6 nm, while maintaining a narrow size distribution. TEM imaging confirmed the spherical morphology of the nanogels formed by the self-assembly of the comb-like polymers, with particle sizes consistent with DLS measurements (Figure 6B). The zeta potential of the HCPT-PLP-TDA 30% nanogel was −20.2 ± 0.3 mV (Figure 6C), suggesting good colloidal stability conferred by electrostatic repulsion.

### 2.5. pH-Responsiveness of Nanogel

**Figure 7:**
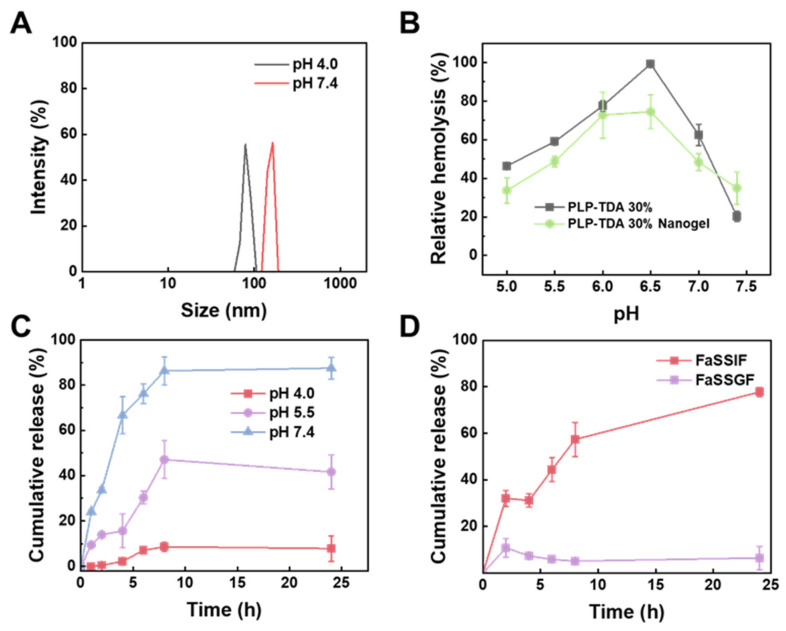
Evaluation of pH-responsive behavior in PLP-TDA 30% nanogels. (**A**) Hydrodynamic size distribution at pH 7.4 and 4.0. (**B**) pH-dependent membrane destabilization activity (relative hemolysis, %) across physiological and acidic pH ranges. (**C**) In vitro HCPT release from PLP-TDA 30% nanogels with different pH values (4.0, 5.5, 7.4). (**D**) Gastrointestinal release simulation: HCPT release in FaSSGF (pH 1.2) and FaSSIF (pH 7.4). Mean ± SD (n = 3).

The pH-responsiveness of the prepared PLP-TDA 30% nanogels was evaluated through pH-dependent particle size changes, membrane destabilization activity, and in vitro drug release profiles. As depicted in Figure 7A, the hydrodynamic size of PLP-TDA 30% nanogels exhibited significant pH sensitivity. At physiological pH (7.4), the mean particle size was 164.0 nm, likely attributable to electrostatic repulsion from deprotonated carboxyl groups promoting extended chain conformations. Under acidic conditions (pH 4.0), protonation of carboxyl groups reduced charge density and enhanced hydrophobicity, triggering chain compaction and a dramatic size reduction to 78.8 nm. These conformational transitions directly correlated with membrane-destabilizing activity. Figure 7B demonstrates that PLP-TDA 30% nanogels retained pH-responsive membrane destabilization behavior, although with reduced responsiveness compared to the free PLP-TDA 30% polymer. This attenuation is potentially due to physical crosslinking during nanogel formation restricting the polymer’s coil-to-globule conformational change [34]. Nevertheless, the nanogels achieved over 70% hemolysis within the pH 6.0–6.5 range, typical of the duodenum and jejunum, suggesting potential for precise modulation of intestinal cell membrane permeability.

Furthermore, in vitro HCPT release from PLP-TDA 30% nanogels was assessed across different pH buffers (Figure 7C). Rapid drug release occurred at pH 7.4, reaching 86.3 ± 6.2% cumulative release within 8 h, followed by sustained release achieving 87.5 ± 4.8% within 24 h. In contrast, release rates were significantly slower under acidic conditions: cumulative release decreased to 41.6 ± 7.6% at pH 5.5 and only 8 ± 6% at pH 4.0 after 24 h. Gastrointestinal simulations using fasted-state simulated fluids (FaSSGF, pH 1.2 and FaSSIF, pH 7.4) confirmed this acid-resistant release profile. As seen in Figure 7D, merely 7 ± 5% HCPT was released in FaSSGF versus 77.7 ± 2.0% in FaSSIF. This behavior originates from pH-triggered conformational changes: protonation under acidic (gastric) conditions contracts the nanogel network, restricting drug diffusion, while deprotonation under neutral (intestinal) conditions reduces diffusion resistance, facilitating release. This mechanism is consistent with the observed pH-dependent size changes (Figure 7A). The resulting “acid-inhibited/neutral-triggered” release profile offers gastric protection and targeted intestinal delivery, potentially enhancing the oral bioavailability of poorly soluble drugs like HCPT.

### 2.6. Cellular Uptake

**Figure 8:**
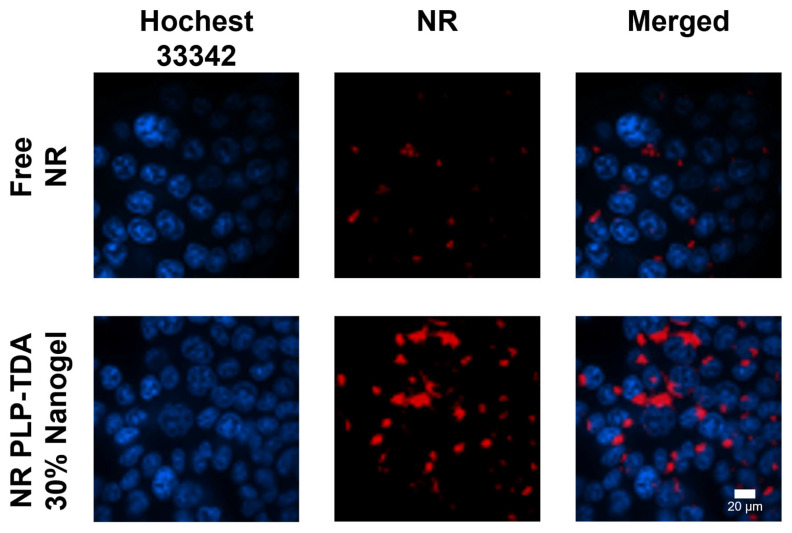
Confocal microscopy images showed the intracellular delivery of the free NR and NR-loaded PLP-TDA 30% nanogel. Scale bar: 20 μm.

To evaluate cellular uptake of PLP-TDA 30% nanogels, Nile Red (NR), a red-fluorescent probe, was encapsulated using the same protocol as for HCPT. HCT116 cells (human colorectal carcinoma line) were incubated for 2 h with free NR and NR-loaded PLP-TDA 30% nanogels at equivalent NR concentrations (5 μg/mL). As shown in Figure 8, minimal punctate red fluorescence was observed in cells treated with free NR, whereas the NR nanogel group exhibited substantially enhanced intracellular fluorescence. This demonstrates the nanogels’ ability to significantly improve cellular internalization compared with the free drug [35]. The enhanced uptake likely stems from two mechanisms: (1) enhanced interactions between the polymer’s grafted side chains and cell membranes and (2) recognition by scavenger receptors—glycoproteins overexpressed on HCT116 cells—potentially facilitated by the nanogel’s negative zeta potential (−20.2 mV).

### 2.7. In Vitro Cytotoxicity

**Figure 9:**
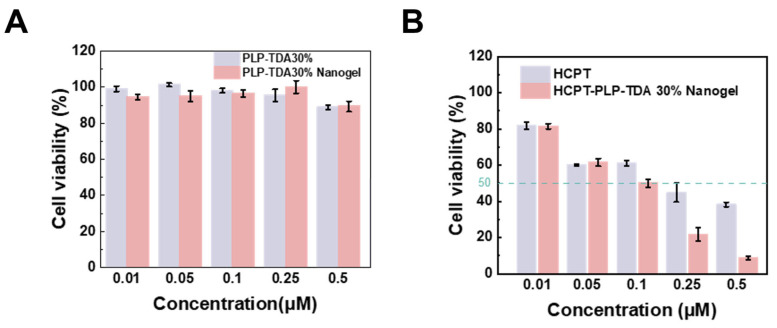
In vitro cytotoxicity evaluation. (**A**) Concentration-dependent viability of HCT116 cells treated with PLP-TDA 30% polymer and nanogel without drug loading. (**B**) Potency of free HCPT and HCPT-PLP-TDA 30% nanogel against HCT116 cells. Mean ± SD (n = 3).

The in vitro cytotoxicity of both the PLP-TDA 30% polymer and its corresponding nanogel was evaluated. As shown in Figure 9A, excellent cytocompatibility was observed across a wide concentration range, with cell viability consistently exceeding 85%, indicating favorable biocompatibility.

The therapeutic efficacy of HCPT-loaded PLP-TDA 30% nanogel was rigorously assessed via concentration-dependent cytotoxicity assays against HCT116 cells. Figure 9B demonstrates that HCPT-loaded nanogel exhibited potent anti-proliferative activity, achieving a significantly lower half-maximal inhibitory concentration (IC_50_ = 0.14 μM) compared with free HCPT (IC_50_ = 0.28 μM). This twofold enhancement in cytotoxic potency underscores the critical contribution of nanogel-mediated delivery. The superior efficacy directly correlates with the established pH-responsive drug release profile and enhanced cellular uptake, demonstrating that these nanogels not only improve drug bioavailability but also significantly augment tumor cell killing [36,37].

## 3. Conclusions

In this study, a series of pH-responsive comb-like polymers were successfully synthesized by grafting alkylamines of varying carbon chain lengths (C_10_, C_14_, and C_18_) onto a PLP backbone with systematic control over grafting density. The results demonstrate that both alkyl chain length and grafting density exerted a profound influence on the pH-dependent conformational transitions, hydrodynamic sizes, and membrane-disrupting capabilities of the polymers. Nanogels fabricated from these comb-like polymers were subsequently evaluated, among which the formulation with TDA grafted at 30% density exhibited optimal hydrophobic interactions and network compactness. This optimized nanogel achieved the highest drug-loading capacity for HCPT along with ideal particle size and colloidal stability. Notably, under simulated gastric conditions (pH 1.2), the system sustained limited drug release (7 ± 5% over 24 h), effectively shielding HCPT from acid degradation. In contrast, under neutral intestinal pH (7.4), a rapid and extensive drug release profile was observed (77.7 ± 2.0% within 24 h), indicating promising intestinal-targeted release behavior. Furthermore, the HCPT-loaded PLP-TDA 30% nanogels significantly enhanced cellular internalization and promoted cytoplasmic delivery, resulting in a twofold increase in cytotoxic activity against colorectal cancer cells. Collectively, these findings highlight the potential of hydrophobically modified pH-responsive nanogels. To pave the way for future translation, further investigations will focus on key parameters including the residual DMSO amount, polymer isoelectric point, thermal sterilization capabilities, and muco-adhesion properties, alongside a detailed understanding of the in vivo fate of the polymers.

## 4. Materials and Methods

### 4.1. Materials

Hydrochloric acid (HCl, 99.8%) and chloroform (CHCl_3_, 98%) were purchased from Nanjing Wanqing Chemical & Glass Instrument Co., Ltd. (Nanjing, China). Sodium hydroxide (NaOH, AR, Analytical Reagent) was obtained from Xilong Scientific Co., Ltd. (Shantou, China). Dimethyl sulfoxide (DMSO, 99.7%) and *N*,*N*-dimethylformamide (DMF, 99.7%) were purchased from Shanghai Energy Chemical Co., Ltd. (Shanghai, China). n-decylamine (NDA, 98%), tetradecylamine (TDA, 96%), octadecylamine (ODA, 98%), sodium dihydrogen phosphate dihydrate (NaH_2_PO_4_·2H_2_O, AR), disodium hydrogen phosphate dodecahydrate (Na_2_HPO_4_·12H_2_O, 99%), citric acid monohydrate (C_6_H_8_O_7_·H_2_O, 99.5%), sodium citrate dihydrate (C_6_H_5_Na_3_O_7_·2H_2_O, 99%), and 4-dimethylaminopyridine (DMAP, 99.8%) were all purchased from Shanghai Aladdin Biochemical Technology Co., Ltd. (Shanghai, China). 10-hydroxycamptothecin (HCPT, 98%) was acquired from Shanghai Titan Scientific Co., Ltd. (Shanghai, China). Dicyclohexylcarbodiimide (DCC, 99.8%) was purchased from Shanghai Energy Chemical Co., Ltd. (Shanghai, China), with all reagents used directly without further purification. McCoy’s 5A medium, fetal bovine serum (FBS) and penicillin–streptomycin were purchased from Nanjing Xunbei Biotechnology Co., Ltd. (Nanjing, China). 3-(4,5-dimethylthiazol-2-yl)-2,5-diphenyltetrazolium bromide (MTT, 98%) and Hoechst staining solution were both purchased from Shanghai Biyuntian Biotechnology Co., Ltd. (Shanghai, China).

### 4.2. Synthesis of pH-Responsive Comb Polymers

PLP was conjugated with alkylamines, specifically NDA, TDA, and ODA, at varying substitution degrees to synthesize comb-like polymers. The conjugation was achieved via DCC/DMAP-mediated amide coupling chemistry [38]. The resulting polymers, denoted as PLP-NDA 10%, PLP-NDA 20%, PLP-NDA 30%, PLP-TDA 10%, PLP-TDA 20%, PLP-TDA 30%, PLP-ODA 10%, PLP-ODA 20%, and PLP-ODA 30%, were prepared by grafting the hydrophobic alkyl chains onto the carboxylic acid groups along the backbone of the same parent PLP. Consequently, all polymers shared an identical PDI. The numerical suffix indicates the stoichiometric molar percentage of alkylamine relative to the pendant carboxylic acid groups of PLP (Table S1), following previously published methods [10,21]. Briefly, a specified amount of NDA, TDA, or ODA (Table S1) was dissolved in 1 mL of chloroform. Separately, 1 g of PLP and 0.2 g of DMAP were dissolved in 20 mL of a DMSO:DMF mixture (1:3 *v*/*v*). The two solutions were combined, and a solution of DCC in 3.4 mL of DMF was added dropwise. The reaction mixture was stirred for 48 h at room temperature to ensure completion. The crude mixture was then vacuum-filtered to remove insoluble impurities. Subsequently, 20 mL of a 5% (*w*/*v*) NaOH solution in ethanol was added to the filtrate. The polymer was precipitated by rapid addition of diethyl ether (five times the solution volume). The precipitate was collected, dissolved in deionized water, and reprecipitated by adding 0.2 M HCl. The precipitated polymer was redissolved in 0.2 M NaOH, and the solution was filtered. This precipitation–filtration–redissolution cycle (using HCl for precipitation and NaOH for dissolution) was repeated twice to remove inorganic salts and residual organic reagents. Finally, the polymer was purified by dialysis against distilled water for 3–4 days using a membrane with a molecular weight cutoff (MWCO) of 12–14 kDa. The dialyzed solution was lyophilized to afford the target pH-responsive comb polymers.

### 4.3. Structural Characterization

The acid-precipitated polymer was dissolved in d_6_-DMSO, and ^1^H NMR spectra for were acquired at room temperature on a 400 MHz NMR spectrometer (V-700, Bruker, Billerica, MA, USA) for structural analysis. The grafting degree (n%)—defined as the actual molar percentage of grafted alkyl chain relative to the side-chain carboxylic groups on the PLP backbone—was calculated from the ratio of integral values at 0.77–0.91 ppm (alkyl CH_3_ protons) to 7.45~7.64 ppm (aromatic protons). FT-IR spectra were recorded on an infrared spectrometer (IS 5, NICOLET, Madison, WI, USA) over the range of 400–4000 cm^−1^ [39].

### 4.4. Turbidity Measurement

Polymer solutions were prepared in phosphate or citrate buffer (100 mM) at a final concentration of 0.5 mg/mL at specific pHs (pH 3.5–7.4) and allowed to equilibrate for 48 h. The transmittance of the polymer solutions at different pHs was measured using a UV-Vis spectrophotometer (V-700, JASCO, Tokyo, Japan).

### 4.5. CAC Determination

pH-responsive comb polymers were dissolved in deionized water to prepare a concentration gradient (0.01~10 μg/mL), filtered through a 0.22 μm membrane, and analyzed using a DLS instrument (ZS90, Malvern, Malvern, UK) at 25 °C with a scattering angle of 90° (11 scans per sample). The CAC was determined as the concentration corresponding to the inflection point in the plot of scattered light intensity versus polymer concentration [40].

### 4.6. pH-Responsive Membrane Destabilization Assay

Polymer solutions were dissolved in 0.1 M phosphate buffer (PBS, pH ≥ 5.5) or citrate buffer (CBS, pH < 5.5) to a final concentration of 0.5 mg/mL. Intact defibrinated sheep red blood cells (RBCs) were washed at least four times with 150 mM of NaCl solution (equivalent to 0.9% NaCl) and resuspended in the polymer buffer to a final concentration of 2 × 10^8^ cells/mL. Negative and positive controls were prepared by mixing RBCs with sterile NaCl solution and deionized water, respectively. All samples were incubated at 37 °C with shaking at 120 rpm for 1 h, then centrifuged at 4000 rpm for 4 min (64R, Allegra, Brea, CA, USA). The absorbance of the supernatant at 540 nm was measured using a microplate reader (Nivo, PerkinElmer, Helsinki, Finland) to quantify hemoglobin release. The relative membrane destabilization rate was calculated by normalizing the test sample absorbance to the positive control (100% destruction):

(1) $$Membrane\;disruption\left(\%\right)=\frac{{OD}_{b}-{OD}_{c}}{{OD}_{a}-{OD}_{c}}\times 100\%$$

In the formula, ${OD}_{c}$ represents the negative control group, ${OD}_{b}$ represents the experimental group, and$\;{OD}_{a}$ represents the positive control group.

### 4.7. Preparation of Nanogels

Nanogels were synthesized via the nanoprecipitation method. Ten milligrams of pH-responsive comb-like polymers were dissolved in 0.4 mL of DMSO, mixed uniformly, and slowly dropped into 50 mL of vigorously stirred deionized water. The mixture was stirred for 2 h. To load HCPT, the drug and polymer were co-dissolved in DMSO prior to subsequent steps, and all procedures were performed under light protection to prevent photodegradation. The nanogels were dialyzed against distilled water for 3 days using a 12–14 kD_a_ dialysis bag to remove unencapsulated drug.

### 4.8. Particle Size and Zeta Potential Measurement

The hydrodynamic size, the PDI and zeta potential of the nanogels were measured using a Malvern particle size analyzer [41]. Polymers were dissolved in 0.1 M PB buffer (0.5 mg/mL) at different pH values and equilibrated at room temperature for 48 h. Particle size and size distribution was measured at 25 °C with a 90° scattering angle (11 runs per sample). The morphology of the prepared nanogel was observed by TEM (HT7700, Hitachi, Tokyo, Japan).

### 4.9. EE and DL

Ultrafiltration centrifugation was applied to remove the unencapsulated HCPT. The purified nanogel and the supernatant after ultrafiltration was lyophilized and dissolved in DMF. The absorbance of HCPT was measured using a UV-Vis spectrophotometer at 385 nm [42]. The EE and DL were calculated using the following equations:

(2) $$EE\left(\%\right)=\frac{W_{t}-W_{f}}{W_{t}}\times 100$$

(3) $$DL(\%)=\frac{Wt-W_{f}}{W_{NG}}\times 100$$

In the formula, *W_t_* is the total HCPT content, and *W_f_* is denoted as the unloaded HCPT content, and *W_NG_* refers to the nanogel weight.

### 4.10. In Vitro Drug Release

In vitro drug release from nanogels was evaluated under different pH conditions. A 1 mL aliquot of nanogel solution was sealed in a dialysis bag (MWCO 12–14 kDa) and immersed in 10 mL of preheated PBS or citric acid buffer containing 0.5 (*v*/*v*) Tween 20 under continuous shaking at 100 rpm (37 °C) to ensure the sink condition was maintained. At predetermined time intervals, 500 μL of the release medium was withdrawn and replaced with an equal volume of fresh buffer. The concentration of released HCPT was quantified by measuring absorbance at 385 nm using UV-Vis spectroscopy [43].

Drug release profiles were further characterized in biorelevant media: fasted-state simulated gastric fluid (FaSSGF) and fasted-state simulated intestinal fluid (FaSSIF) [44,45,46]. FaSSGF was prepared by dissolving 2.0 g of NaCl in deionized water, adjusting to pH 1.2 with hydrochloric acid, and diluting to a final volume of 1 L. FaSSIF was prepared by dissolving 6.8 g of KH_2_PO_4_ in 500 mL of deionized water, adjusting to pH 7.4 with 0.9 g of NaOH, and diluting to 1 L with deionized water. Release studies in these media followed the aforementioned dialysis method.

### 4.11. Cell Culture

Human colorectal cancer cells (HCT116) were cultured in McCoy’s 5A medium supplemented with 10% (*v*/*v*) FBS and 1% (*v*/*v*) penicillin–streptomycin. HCT116 cells were trypsinized using trypsin-EDTA for 2 min and maintained at 37 °C in a humidified incubator containing 5% CO_2_.

### 4.12. In Vitro Cytotoxicity Assay

In vitro cytotoxicity was evaluated using the MTT assay [47]. HCT116 cells were seeded in 96-well plates at a density of 1 × 10^4^ cells/well and cultured in McCoy’s 5A medium supplemented with 10% fetal bovine serum (FBS) and 1% penicillin/streptomycin for 24 h. After incubation, the medium was replaced with 100 μL of filter-sterilized (0.22 μm) polymer or nanogel solutions and incubated for an additional 24 h. Cells treated with culture medium alone served as the negative control. Following treatment, cells were washed three times with PBS and incubated with 100 μL of serum-free medium containing 0.5 mg/mL of MTT for 4 h. The MTT-containing medium was then carefully removed, and 100 μL of DMSO was added to each well to solubilize the formazan crystals. After shaking at 120 rpm for 15 min at 37 °C, absorbance was measured at 562 nm using a microplate reader.

### 4.13. Cellular Uptake

HCT116 cells were seeded at a density of 1 × 10^5^ cells per well in 20 mm glass-bottom culture dishes and incubated for 24 h. After washing with PBS, cells were treated for 2 h with either free NR or NR-loaded nanogel at a final NR concentration of 10 μg/mL. Following treatment, cells were washed three times with ice-cold PBS and fixed with 4% paraformaldehyde (PFA) in PBS for 15 min at room temperature. Fixed cells were then stained with 10 μg/mL of Hoechst 33342 for 15 min to label nuclei. Cellular imaging was performed using a laser scanning confocal microscope (Nikon Ti2-A, Nikon Corporation, Tokyo, Japan). NR was excited at 555 nm with emission collected at 570–620 nm; Hoechst 33342 was excited at 405 nm with emission at 425–475 nm [48].

### 4.14. Statistical Analysis

Statistical analysis. All data are expressed as mean ± standard deviation (SD). Statistical analysis was performed with Student’s *t*-test. *p* values < 0.05 were considered statistically significant.

## Figures and Tables

**Table 1:** Mean particle size of nanogels during 30-day storage at 4 °C.

	Days	0 d	7 d	14 d	30 d
Average Size (nm)					
PLP-NDA 10% nanogel		58.7 ± 0.7	63.4 ± 5.5	59.6 ± 2.2	60.0 ± 2.3
PLP-NDA 20% nanogel		85.7 ± 3.0	85.2 ± 4.8	80.7 ± 4.0	78.4 ± 3.5
PLP-NDA 30% nanogel		84.0 ± 4.5	84.4 ± 3.7	83.4 ± 2.6	83.0 ± 3.9
PLP-TDA 10% nanogel		83.2 ± 0.7	78.7 ± 0.8	79.0 ± 1.3	83.5 ± 0.9
PLP-TDA 20% nanogel		92.0 ± 0.4	82.6 ± 0.3	82.8 ± 1.6	96.7 ± 0.6
PLP-TDA 30% nanogel		105.4 ± 1.8	101.0 ± 2.7	102.8 ± 1.8	105.1 ± 3.2
PLP-ODA 10% nanogel		87.4 ± 6.7	82.4 ± 5.2	88.2 ± 8.8	91.0 ± 5.7
PLP-ODA 20% nanogel		94.4 ± 9.2	101.7 ± 10.8	115.8 ± 11.5	106.3 ± 2.6
PLP-ODA 30% nanogel		165.0 ± 13.2	177.7 ± 41.0	232.6 ± 61.9	252.5 ± 4.3

## Data Availability

Data will be available on request.

## Supplementary Materials

The following supporting information can be downloaded at https://www.mdpi.com/article/10.3390/gels11100806/s1, Table S1: Grafting ratio of alkylamines with different chain length onto PLP. The specific quantities used for synthesis of comb-like polymer are provided; Figure S1: 1H NMR spectra of PLP-NDA polymer samples with different grafting ratios; Figure S2: 1H NMR spectra of PLP-TDA polymer samples with different grafting ratios; Figure S3: 1H NMR spectra of PLP-ODA polymer samples with different grafting ratios; Table S2: pHp and CAC value of PLP and its comb-like derivatives; Table S3: PDI of nanogels during 30 days storage at 4 °C; Table S4: Encapsulation efficiency (EE) and drug-loading capacity (DLC) of camptothecin in nanogels prepared by various polymers.
