# Supplementary material for: pH-Responsive Nanogels from Bioinspired Comb-like Polymers with Hydrophobic Grafts for Effective Oral Delivery
Source: Gels. 2025 Oct 8;11(10):806. doi: 10.3390/gels11100806 (PMC12564256; doi:10.3390/gels11100806)
Supplement: Supplementary file 1 [file gels-11-00806-s001.zip › gels-3880101-supplementary.pdf]

Supplementary Materials

pH-Responsive Nanogels from Bioinspired Comb-like Polymers with Hydrophobic Grafts for Effective Oral Delivery

Qinglong Liu [a], Dewei Ma [a], Haoze Cheng [a], Keke Yang [a], Bo Hou [a], Ziwen Heng [a], Yu Qian [a], Wei Liu [b], Siyuan Chen [a*]

a. Research Institute for Biomaterials, Tech Institute for Advanced Materials Bioinspired Biomedical Materials & Devices Center, College of Materials Science and Engineering, Jiangsu Collaborative Innovation Center for Advanced Inorganic Function Composites, Suqian Advanced Materials Industry Technology Innovation Center, Nanjing Tech University, Nanjing, China.

b. College of Life Science and Technology, Huazhong University of Science and Technology, Wuhan, 430074, China.

*Email: siyuan.chen@njtech.edu.cn

Table S1. Grafting ratio of alkylamines with different chain length onto PLP. The specific quantities used for synthesis of comb-like polymer are provided.

Polymer abbreviation	Side Chain length	Grafting ratio (%)	PLP (g)	NDA (mg)	TDA (mg)	ODA (mg)
PLP-NDA 10%	C_{10}	10	1	56.98	/	/
PLP-NDA 20%	C_{10}	20	1	113.96	/	/
PLP-NDA 30%	C_{10}	30	1	170.90	/	/
PLP-TDA 10%	C_{14}	10	1	/	77.32	/
PLP-TDA 20%	C_{14}	20	1	/	154.65	/
PLP-TDA 30%	C_{14}	30	1	/	231.97	/
PLP-ODA 10%	C_{18}	10	1	/	/	97.66
PLP-ODA 20%	C_{18}	20	1	/	/	195.32
PLP-ODA 30%	C_{18}	30	1	/	/	292.98

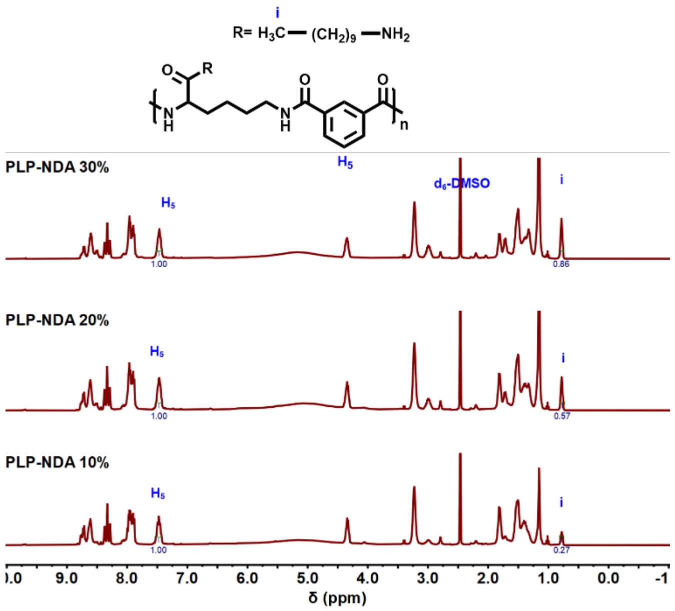

Figure S1. ^{1}H NMR spectra of PLP-NDA polymer samples with different grafting ratios.

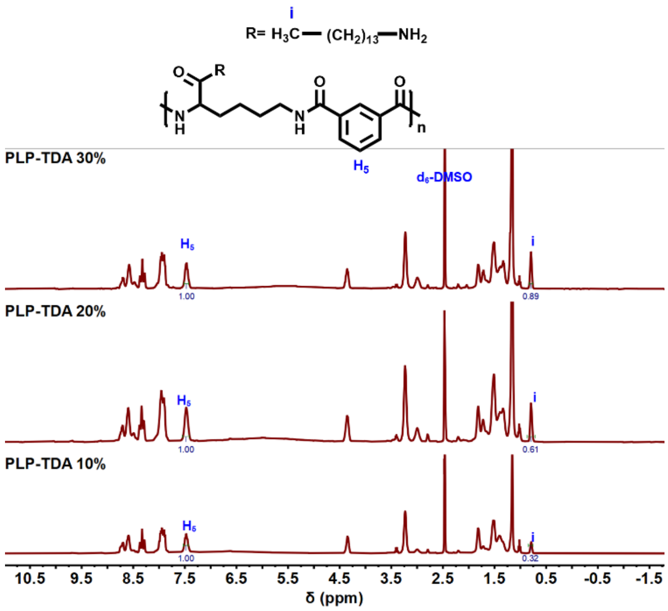

Figure S2. ^{1}H NMR spectra of PLP-TDA polymer samples with different grafting ratios.

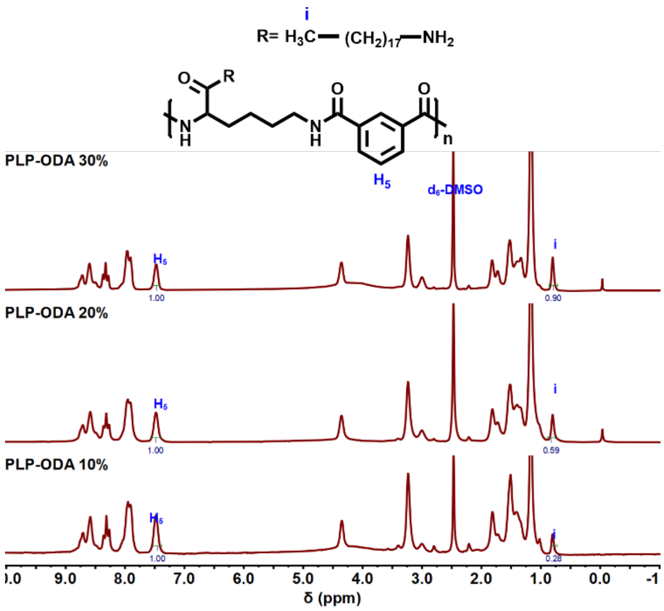

Figure S3. ^{1}H NMR spectra of PLP-ODA polymer samples with different grafting ratios.

Table S2. pH_P and CAC value of PLP and its comb-like derivatives.

	**pH_P**	**CAC (μg/mL)**
PLP	4.0	50.0
PLP-NDA 10%	4.0	40.0
PLP-NDA 20%	4.5	20.0
PLP-NDA 30%	5.0	10.0
PLP-TDA 10%	4.0	30.0
PLP-TDA 20%	4.5	10.0
PLP-TDA 30%	4.5	5.0
PLP-ODA 10%	5.5	5.0
PLP-ODA 20%	5.5	5.0
PLP-ODA 30%	7.4	4.0

Table S3. PDI of nanogels during 30 days storage at 4℃.

Days / PDI	0 d	7 d	14 d	30 d
PLP-NDA 10% nanogel	0.13 ± 0.01	0.20 ± 0.01	0.18 ± 0.01	0.19 ± 0.00
PLP-NDA 20% nanogel	0.08 ± 0.00	0.10 ± 0.01	0.14 ± 0.02	0.12 ± 0.00
PLP-NDA 30% nanogel	0.10 ± 0.00	0.10 ± 0.02	0.09 ± 0.01	0.08 ± 0.02
PLP-TDA 10% nanogel	0.12 ± 0.01	0.20 ± 0.03	0.15 ± 0.03	0.19 ± 0.03
PLP-TDA 20% nanogel	0.10 ± 0.02	0.09 ± 0.00	0.12 ± 0.04	0.28 ± 0.01
PLP-TDA 30% nanogel	0.10 ± 0.01	0.11 ± 0.09	0.12 ± 0.05	0.18 ± 0.02
PLP-ODA 10% nanogel	0.22 ± 0.05	0.27 ± 0.09	0.31 ± 0.01	0.31 ± 0.03
PLP-ODA 20% nanogel	0.22 ± 0.09	0.24 ± 0.03	0.32 ± 0.06	0.21 ± 0.01
PLP-ODA 30% nanogel	0.37 ± 0.01	0.39 ± 0.03	0.49 ± 0.12	0.40 ± 0.21

Table S4. Encapsulation efficiency (EE) and drug loading capacity (DLC) of camptothecin in nanogels prepared by various polymers.

	**EE (%)**	**DLC (%)**
PLP-NDA 10% nanogel	18.81 ± 0.79	1.71 ± 0.07
PLP-NDA 20% nanogel	18.85 ± 0.60	1.71 ± 0.05
PLP-NDA 30% nanogel	34.57 ± 0.24	3.14 ± 0.02
PLP-TDA 10% nanogel	15.76 ± 0.44	1.43 ± 0.04
PLP-TDA 20% nanogel	39.85 ± 9.62	3.62 ± 0.87
PLP-TDA 30% nanogel	47.50 ± 3.12	4.31 ± 0.28
PLP-ODA 10% nanogel	29.11 ± 0.28	2.64 ± 0.02
PLP-ODA 20% nanogel	34.18 ± 3.18	3.10 ± 0.28
PLP-ODA 30% nanogel	38.03 ± 0.35	3.45 ± 0.03
